# 

**Published:** 1915-06-12

**Authors:** 


### Buildings Contemplated and in Progress.

London, W.—The new building of the Margaret Street Hospital for Consumption in Margaret Street, Cavendish Square, has been formally opened. Mr. F. M. Elgood was the architect. The cost was £9,000.

Lower Penn.—The Staffordshire Tuberculosis Committee have selected a site at Lower Penn for a sanatorium.

Norton.—The Stockton Guardians have received sanction to a loan for the purchase of a site for a new infirmary.

Raywell.—At a recent meeting of the East Riding and City of York Joint Sanatorium Provisional Committee at York, the Provisional Committee approved the sketch-plans for the sanatorium at Raywell. Mr. Stamford is the architect. Detailed plans will be prepared and submitted to the Local Government Board for approval.

### Gifts and Bequests.

MR. ALFRED CHARLES WOODHOUSE, of Blandford Forum, Dorset, brewer and wine and spirit merchant, a former Mayor of Blandford, has left £100 to the Blandford Cottage Hospital.

MISS MARY CROFT, of Waterloo, Lancs., formerly of Liscard, Cheshire, has left £300 to the Catholic Blind Asylum, Liverpool; and smaller legacies to a number of other Roman Catholic charities.

MR. THOMAS GROOM BARKER, of Eccles, and of a firm of architects of Manchester, who left estate valued at £14,912 gross, has bequeathed £600 to the Manchester Infirmary; £400 to the St. Mary's Hospital, Manchester; £150 to the Royal School for the Deaf and Dumb, Old Trafford; £150 to the Chethams Hospital; and £50 to Dr. Barnardo's Homes.

### NEW MEDICAL SUPERINTENDENT OF DERBY COUNTY MENTAL HOSPITAL.

DR. MARRIOTT LOGAN ROWELL, the newly appointed medical superintendent of the Derby County Mental Hospital at Mickleover, has found in the assistant medical officership the passport to promotion to the higher post. Graduating B.A., M.B., B.Ch., B.A.O., from Queen's College, Belfast, in 1900, Dr. Rowell in the year 1903 took the M.D. degree of the Royal University of Ireland.

After service as a civil surgeon in the R.A.M.C., he, in 1904, went as second assistant medical officer to the Derby County Asylum, and was in 1907 promoted first assistant medical officer, which post he now vacates on appointment as medical superintendent.

Rates of Subscription : Twelve months, 6/6 ; Six months, 4/- ; Three months, 2/-, post free to any address in the United Kingdom. Abroad, Twelve months, 8/8 ; Six months, 5/- ; Three months, 2/6, post free.—Address The Subscription Dept., "The Hospital," The Hospital Building, 28/29 Southampton Street, Strand, London, W.C.

